# Genotypic effect of *ahFAD2* on fatty acid profiles in six segregating peanut (*Arachis hypogaea* L) populations

**DOI:** 10.1186/1471-2156-14-62

**Published:** 2013-07-17

**Authors:** Noelle A Barkley, Thomas G Isleib, Ming Li Wang, Roy N Pittman

**Affiliations:** 1 USDA ARS Plant Germplasm Resources Conservation Unit, 1109 Experiment Street, Griffin, GA 30223, USA; 2 Department of Crop Science, North Carolina State University, Box 7629, Raleigh, NC 27695-7629, USA

**Keywords:** Real-time PCR, Gas chromatography, *ahFAD2*, High oleic peanuts, Fatty acids, Segregation ratios

## Abstract

**Background:**

Fatty acid composition of oil extracted from peanut (*Arachis hypogaea* L.) seed is an important quality trait because it may affect the flavor and shelf life of resulting food products. In particular, a high ratio of oleic (C18:1) relative to linoleic (C18:2) fatty acid (O/L ≥ 10) results in a longer shelf life. Previous reports suggest that the high oleic (~80%) trait was controlled by recessive alleles of *ahFAD2A* and *ahFAD2B*, the former of which is thought to have a high frequency in US runner- and virginia-type cultivars. Functional mutations, G448A in *ahFAD2A* and 442insA in *ahFAD2B* eliminate or knock down desaturase activity and have been demonstrated to produce peanut oil with high O/L ratios. In order to employ marker assisted selection (MAS) to select a high oleic disease resistant peanut and to evaluate genotypic and phenotypic variation, crosses were made between high oleic (~80%) and normal oleic (~50%) peanuts to produce segregating populations.

**Results:**

A total of 539 F_2_ progenies were randomly selected to empirically determine each *ahFAD2* genotype and the resulting fatty acid composition. Five of the six crosses segregated for the high oleic trait in a digenic fashion. The remaining cross was consistent with monogenic segregation because both parental genotypes were fixed for the *ahFAD2A* mutation. Segregation distortion was significant in *ahFAD2A* in one cross; however, the remaining crosses showed no distortion. Quantitative analyses revealed that dominance was incomplete for the wild type allele of *ahFAD2*, and both loci showed significant additive effects. Oleic and linoleic acid displayed five unique phenotypes, based on the number of *ahFAD2* mutant alleles. Further, the *ahFAD2* loci did exhibit pleiotropic interactions with palmitic (C16:0), oleic (C18:1), linoleic (C18:2) acids and the O/L ratio. Fatty acid levels in these progeny were affected by the parental genotype suggesting that other genes also influence fatty acid composition in peanut. As far as the authors are aware, this is the first study in which all of the nine possible *ahFAD2* genotypes were quantitatively measured.

**Conclusions:**

The inheritance of the high oleic trait initially was suggested to be controlled by dominant gene action from two homoeologous genes (*ahFAD2A* and *ahFAD2B*) exhibiting complete recessivity. Analyzing the *ahFAD2* genotypes and fatty acid compositions of these segregating peanut populations clearly demonstrated that the fatty acid contents are quantitative in nature although much of the variability in the predominant fatty acids (oleic, linoleic, and palmitic) is controlled by only two loci.

## Background

Peanut (*Arachis hypogaea* L., *2n* = *4x* = 40) is a major oilseed crop worldwide. Regular dietary intake of its seeds provides a significant source of protein, folate, tocopherol, phytosterols, polyphenolics such as resveratrol, fiber, and edible oil. Regular consumption of peanuts has been demonstrated to have positive impacts on human health [1-3]. Chemical and epidemiological studies suggest that the nutritional properties of peanuts are favorable due to the fatty acid profiles (containing a minimum of 40% monounsaturated fatty acids), a high quality protein ratio score, and a source of naturally occurring folate [4]. A diet rich in monounsaturated fats containing high levels of fatty acids such as oleic acid (C18:1) has been associated with a reduction in systolic blood pressure [5], reduction of triacylglycerol [6], guarding low density lipoproteins (LDL) from oxidative modification [7], reduction of LDL and total cholesterol in hypercholesterolics [8], helping maintain good cholesterol levels known as high density lipoproteins (HDL), reduction of blood glucose levels in type II diabetes [9], and slowing down atherosclerosis [10].

Cultivated peanut is an allotetraploid and typically contains about 50% oil in the seeds. The majority (~80-90%) of the extracted oil is composed of three primary fatty acids: palmitic (C16:0), oleic (C18:1), and linoleic (C18:2). The flavor, stability, shelf life, and nutritional quality of peanut and peanut products are dependent on the fatty acid composition (ratio of saturated, monounsaturated, and polyunsaturated lipids) of the extracted oil [11-13]. All oils are prone to oxidation over time, which leads to noxious odors and off flavors in stored peanut products. Further, oxidized lipids have been associated with atherosclerosis [14]. Oxidative rancidity is more prevalent among oils with high levels of polyunsaturated fatty acids due to the carbon double bonds that degrade over time producing acids, aldehydes, ketones and hydrocarbons [12]. Peanut that has a high percentage of oleic acid (monounsaturated ω-9 fatty acid) and a low percentage of linoleic acid (polyunsaturated ω-6 fatty acid) in the oil is less susceptible to rancidification which results in a longer shelf life for stored food products. Therefore, an emphasis from manufacturers and consumers has been placed on breeding peanut with high oleic acid and low linoleic acid.

Due to the efforts of the peanut breeding program in Florida in the 1980s, the first high oleic peanuts F435-2-1 and F435-2-2 were identified which had oleic levels (~80%) comparable to that found in olive (*Olea europaea* L.) oil [15]. The high oleic acid seed oil trait in peanut (*Arachis hypogaea* L.) was originally designated *Ol*_*1*_-*ol*_*1*_ and *Ol*_*2*_-*ol*_*2*_ by Moore and Knauft [12]. The associations of *ol*_*1*_ and *ol*_*2*_ with the A and B genomes of *Arachis hypogaea* were not specified in the genes’ original descriptions, but more recent molecular literature renames the loci *ahFAD2A* and *ahFAD2B*. The inheritance of this desirable phenotype was later demonstrated to be controlled by two key homozygous recessive mutations in two homoeologs *ahFAD2A* and *ahFAD2B*, which encode microsomal oleoyl-PC desaturase, also known as Δ^12^ fatty acid desaturase [12,13,16,17]. Microsomal oleoyl-PC desaturases are responsible for catalyzing the conversion of oleate to linoleate [18] by the addition of a second double bond in the hydrocarbon chain which generates a polyunsaturated fatty acid from a monounsaturated fatty acid [19]. The mutations necessary for the high oleic phenotype found in these homoeologous genes, G448A (D150N) in *ahFAD2A* and 442insA in *ahFAD2B*, were shown to result in a loss of functional enzyme activity or significantly reduced mRNA transcript levels, respectively [13,16,20]. Both of the mutations (G448A and 442insA) in *ahFAD2* affect the histidine motifs which are involved in the metal ion complex required for oxygen reduction [10,20]. Homozygous recessive mutations in both *ahFAD2A* and *ahFAD2B* (G448A and 442insA) are necessary for the high oleate phenotype; whereas, the normal oleate phenotype can be produced from a single homoeolog expressing a functional enzyme.

Studies following the discovery of the F435 high oleic peanuts also established that the trait segregated either in a digenic (15:1) or monogenic (3:1) fashion now known to be dependent on the normal oleic parental genotype (*ol*_*1*_*ol*_*1*_*Ol*_*2*_*Ol*_*2*_ or *Ol*_*1*_*Ol*_*1*_*Ol*_*2*_*Ol*_*2*_) [12,16]. In most normal-oleic U.S. runner- and virginia-type cultivars (predominantly *A*. *hypogaea* subsp. *hypogaea* var. *hypogaea*), one of the mutant alleles, *ahFAD2A*, often is fixed in the homozygous state, so that the high-oleic characteristic behaves as if controlled by a single gene pair. However, Lόpez et al. (2001) found somewhat more complex inheritance of the trait in crosses involving Spanish-type (*A*. *hypogaea* subsp. *fastigiata* Waldron var. *vulgaris* Harz) parents [21] in that some of the populations did not conform to monogenic or digenic segregation patterns.

Although numerous high oleic cultivars have been developed whose pedigrees include F435 as the high oleic donor, elite high oleic cultivars are still needed to thwart disease and abiotic challenges that exists in different environments [22]. Due to shelf life stability and health benefits, some countries (i.e.- Australia, Argentina, South Africa, Israel) are mostly or solely producing high oleic peanut products for consumption. Recently, efforts have been placed on producing high oleic peanuts with enhanced introgressed traits. Breeding multiple traits of interest, such as nematode resistance or other quality traits, into high oleic cultivars can be time consuming due to the generation time, phenotyping for the traits of interest, and maintaining large populations that are needed to obtain desired trait stacking. One of the main hurdles of breeding programs is purging unwanted phenotypes and genotypes from the segregating populations in early stages and rapidly identifying the trait(s) of interest. This can potentially be overcome by selecting individuals with desired genotypes at key loci by using molecular markers, a process known as marker-assisted selection (MAS). This is most efficient if heterozygous and homozygous states can be distinguished at all the loci of interest. Previously, real-time PCR assays have been developed to genotype peanuts for the *ahFAD2* alleles [23,24] that can indeed detect all of the nine possible genotypes produced in segregating populations. Therefore, the objectives of this research were to (a) to implement *ahFAD2* and nematode resistance markers in MAS for the development of a high oleic nematode resistant peanut lines, (b) determine the *ahFAD2* genotypes and fatty acid profiles in segregating peanut populations to establish a link between each genotype and the resulting fatty acid composition, and (c) determine *ahFAD2* gene action on fatty acid composition by variance analysis.

## Results & discussion

### Fatty acid composition

**Figure 1:**
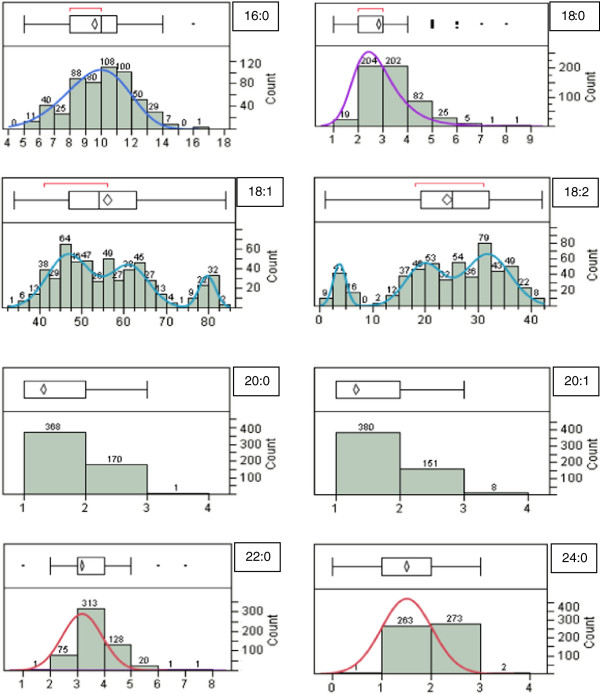
**Histograms of the frequency distribution of the eight collected fatty acids from all six populations.** (Fatty acid values were rounded to the nearest whole number). Ten different continuous fit curves were tested for each histogram and the best fit based on Akaike’s Information Criterion (AIC) was chosen for each histogram. The x-axis shows the range in percentage of each fatty acid collected and the y-axis represents the number of individuals in the F_2_ population.

Fatty acid profiles were collected on all of the parents and 539 F_2_ progeny produced from six crosses (Figure 1). Fitted curves were either normally distributed (C16:0, C22:0, C24:0), skewed (C18:0), or multimodal (C18:1, C18:2). No curves were generated for arachidic (20:0) or gadoleic (20:1) fatty acids because the majority of the F_2_ progeny contained only 1-3% of these fatty acids. Originally, fatty acids in peanut were suggested to be under polygenic control until the discovery and elucidation of the high oleic trait [25]. Later, the inheritance of the high oleic trait initially was suggested to be controlled by dominant gene action from two homoeologous genes (*ahFAD2A* and *ahFAD2B*) exhibiting complete recessivity [12,13]. Once the large effects of *ahFAD2A* and *ahFAD2B* were identified, the remaining phenotypic variation must be ascribed to the effects of the environment and genes with small cumulative effects. Due to the continuous variation observed in this data, fatty acid composition is likely to be polygenically inherited. This is consistent with a previous study which evaluated oleic and linoleic acid contents among progeny derived from backcrossing [25].

**Figure 2:**
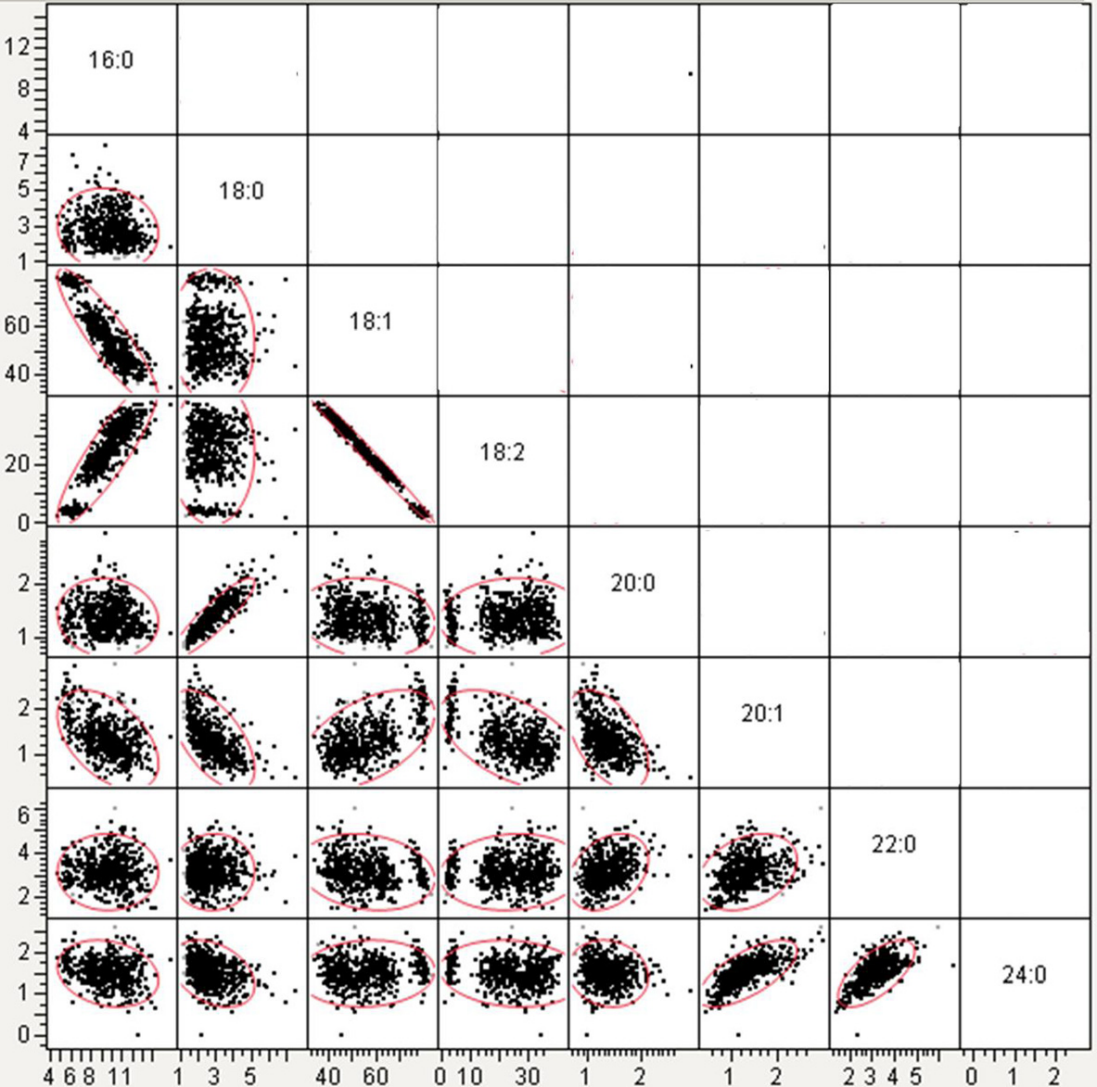
Pairwise comparisons of the correlations among the eight collected fatty acids.

Significant correlations between oleic acid and other fatty acids commonly detected in peanut have been previously reported [11,25-27]. Therefore, pair-wise correlation coefficients were calculated among all the collected fatty acids to determine if these fatty acids vary together or independently in the entire population (Figure 2; Table 1). Pooling the fatty acid data from Crosses 17, 19, 21, 25, 27, and 28 showed significant negative correlations between oleic acid and palmitic acid [16:0] (r = -0.8945, P<0.0001), as well as, oleic and linoleic acid [C18:2] (r = -0.9922, P<0.0001). A significant positive correlation was also revealed between oleic and gadoleic acid [C20:1] (r = 0.5227, P<0.0001). This suggests that the *ahFAD2* genotype may influence the level of oleic, linoleic, palmitic, and gadoleic fatty acids. Furthermore, pleiotropic effects are involved in determining fatty acid composition. Arachidic (C20:0) and behenic (C22:0) fatty acids also correlated with oleic with statistical significance; however, the coefficient of determination (r^2^), which reflects the percentage of variance in oleic acid that can be explained by the variance in another fatty acid, was less than or equal to 3.5%. Pair-wise correlations revealed that fatty acids besides oleic acid were also significantly correlated. Stearic acid (C18:0) was positively and negatively correlated, respectively with arachidic acid [C20:0] (r = 0.8962; P<0.0001) and gadoleic acid [C20:1] (r = -0.5942, P<0.0001). Lignoceric acid (C24:0) was positively correlated with gadoleic [C20:1] (r = 0.6881, P<0.0001) and behenic [C22:0] (r = 0.6657, P<0.0001) fatty acids.

**Table 1:** Pairwise correlations among the eight collected fatty acids

	**16**:**0**	**18**:**0**	**18**:**1**	**18**:**2**	**20**:**0**	**20**:**1**	**22**:**0**	**24**:**0**
16:0	1.0000							
18:0	−0.0923	1.0000						
	P = 0.0322							
18:1	−0.8945	−0.0710	1.0000					
	P < 0.0001	P=0.0996						
18:2	0.8857	0.0134	−0.9922	1.0000				
	P < 0.0001	ns	P < 0.0001					
20:0	−0.0909	0.8962	−0.1347	0.0624	1.0000			
	P = 0.0349	P < 0.0001	P = 0.0017	ns				
20:1	−0.4869	−0.5942	0.5227	−0.5285	−0.5060	1.0000		
	P<0.0001	P<0.0001	P<0.0001	P<0.0001	P<0.0001			
22:0	0.0006	0.0323	−0.1885	0.1032	0.3298	0.3125	1.0000	
	ns	ns	P<0.0001	P=0.0165	P<0.0001	P<0.0001		
24:0	−0.2037	−0.3561	0.0829	−0.1264	−0.1203	0.6881	0.6657	1.0000
	P<0.0001	P<0.0001	ns	P = 0.0033	P = 0.0052	P<0.0001	P<0.0001	

### ahFAD2 Genotypes in segregating peanut populations

The parents, F_1’s_, and 539 F_2_ progeny were all genotyped for *ahFAD2A* and *ahFAD2B* in order to empirically determine a fatty acid profile for each *ahFAD2* genotype. All possible *ahFAD2* genotypes were detected in the larger populations (17, 19, 25, & 28). Only seven (Cross 21) and eight (Cross 27) *ahFAD2* genotypes were detected in the smaller populations (Additonal file 1: Table S1). Chi square analysis was employed to evaluate if the F_2_ progenies were segregating in a digenic 15:1 or mongenic 3:1 fashion for the high oleic phenotype (Table 2). Both of the parents of Cross 25 were determined by *ahFAD2* genotyping to be fixed (homozygous recessive) for the *ahFAD2A* mutation; whereas, the parents of the remaining crosses were homozygous wild type crossed by a high oleic genotype. Therefore, as expected, three of the four crosses with 125 F_2_ progeny were consistent with a 15:1 segregation ratio while cross 25 conformed to a 3:1 ratio. None of these crosses had segregation ratios that did not fit the expected ratios as seen in a previous study [21]. Further, segregation distortion from Mendelian expectations was evaluated for all the F_2_ progenies and for each individual cross (Table 3). Overall, segregation distortion was a relatively minor effect in that only a single cross (Cross 17) showed statistically significant (P ≤ 0.05) distortion at a single locus (*ahFAD2A*). Neither *ahFAD2A* nor *ahFAD2B* was distorted in any of the other crosses or when evaluating the total population. Distortion in loci can be caused by differential representation of distorted genotypes in gametes prior to fertilization or viability differences in zygotes after fertilization [28]. Segregation distortion of molecular markers in cultivated peanut has been reported previously [29-31].

**Table 2:** Segregation analysis of the high oleic trait

**Cross ID**	**Cross**	**Range of O/L ratio in the F**_**2**_**progeny**	**Observed normal oleic**	**Observed high oleic**	**Expected normal (15:1)**	**Expected high(15:1)**	**χ**^**2**^**(15:1)**	**Expected normal(9:7)**	**Expected high(9:7)**	**χ**^**2**^**(9:7)**	**Expected normal(3:1)**	**Expected high(3:1)l**	**χ**^**2**^**(3:1)**
17	Florida 07^a^/*A*. *hypogaea* var. *hirsuta*	0.85-43.58	122	3	117.188	7.8125	2.54	70.3125	54.6875	85.18***	93.75	31.25	32.86***
19	York/*A*. *hypogaea* var. *peruviana*	0.93-61.28	113	12	117.188	7.8125	1.86	70.3125	54.6875	57.86***	93.75	31.25	15.00***
21	Chico^c^/Florida 07	0.85-4.72	18	0	16.875	1.125	2.50	10.125	7.875	12.28***	13.5	4.5	4.74*
25	Tifguard^b^/York	1.14-37.61	84	41	117.188	7.8125	145.88***	70.3125	54.6875	5.65*	93.75	31.25	3.65
27	Florida 07^a^/*A*. *hypogaea* var. *peruviana*	0.92-23.47	20	1	19.6875	1.3125	0.54	11.8125	9.1875	11.44***	15.75	5.25	3.57
28	Chico^c^/York	0.92-39.47	116	9	117.188	7.8125	0.06	70.3125	54.6875	66.38***	93.75	31.25	20.18***

^a^ = [37].

^b^ = [32].

^c^ = [36].

*, **, *** = difference is statistically significant at 0.05, 0.01, and 0.001 probability level, respectively.

**Table 3:** **Test for segregation distortion in*ahFAD2***

**Cross ID**	**Cross**	**Observed Ol**_**1**_**Ol**_**1**_	**Observed Ol**_**1**_**ol**_**1**_	**Observed ol**_**1**_**ol**_**1**_	**χ**^**2**^	**Observed Ol**_**2**_**Ol**_**2**_	**Observed Ol**_**2**_**ol**_**2**_	**Observed ol**_**2**_**ol**_**2**_	**χ**^**2**^
17	Florida 07^a^/*A*. *hypogaea* var. *hirsuta*	43	50	31	6.41*	37	61	24	2.79
19	York/*A*. *hypogaea* var. *peruviana*	25	64	36	2.05	38	49	37	5.28
21	Florida 07^a^/A. hypogaea var. peruviana	3	13	2	4.25	6	6	6	1.14
25	Tifguard^b^/York	0	0	125	0.00	24	60	41	4.72
27	Chico^c^/Florida 07^a^	3	10	8	2.5	9	7	5	2.98
28	Chico^c^/York	36	59	30	0.93	37	62	26	1.94
	Total Population	110	196	107	1.07	151	245	139	4.08

^a^ = [37].

^b^ = [32].

^c^ = [36].

*, **, *** = difference is statistically significant at 0.05, 0.01, and 0.001 probability level, respectively.

### Linking phenotypes and genotypes

**Figure 3:**
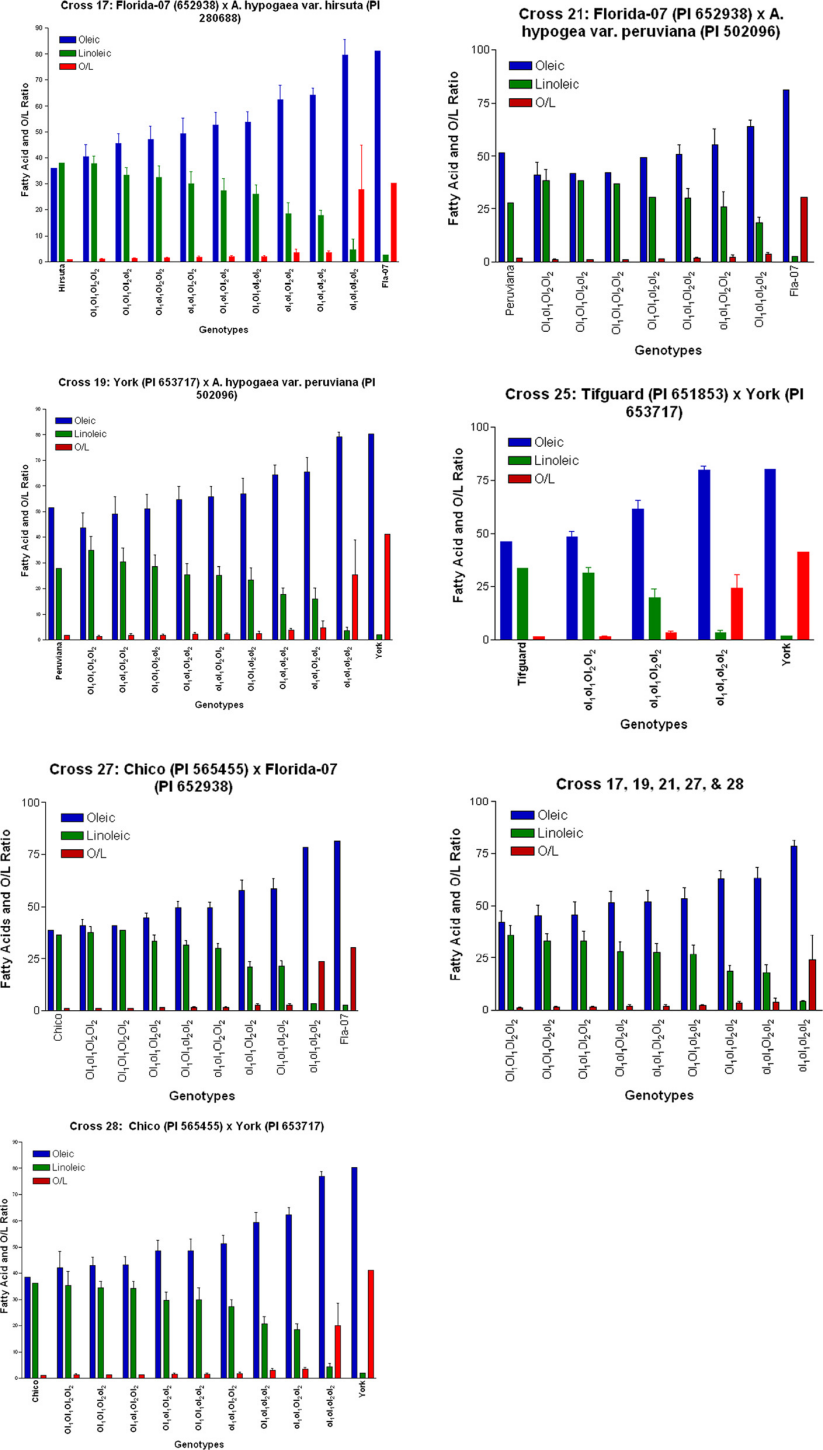
**Mean oleic, linoleic, and O/L ratio of each genotype for each cross and all crosses pooled from the total population that segregated in a 15:1 fashion.** Cross 25 segregating in a 3:1 fashion; whereas, the remaining crosses were consistent with 15:1 segregation. The high and normal oleic parent are also included.

A broad range of oleic acid (C18:1) content was detected from the *ahFAD2* genotypes in the entire population varying from 34.15 to 84.10%; whereas, linoleic acid (C18:2) ranged from 1.31 to 33.08% (Additional file 1: Table S1, Figure 3). As expected, the lowest amount of oleic acid was found in homozygous dominant genotypes, while the highest amounts were in the homozygous recessive genotypes. The O/L ratio varied from 0.85 to 61.28. Palmitic acid ranged from 5.38 to 15.87%; however, the high oleic lines contained significantly less palmitic acid than the normal oleic lines. Stearic acid ranged from 1.13 to 7.59% in the population. Arachidic acid (0.76-2.94%), gadoleic acid (0.5-2.93%), behenic (1.14-6.63%) and lignoceric acid (0-2.74%) contents were detected in these progenies; but, their percentages were fairly small compared to the other fatty acids. Although up to 12 fatty acids have been previously reported as detectable in peanuts, only three (palmitic, oleic, and linoleic) comprise the majority (~90%) of the fatty acids found in peanut oil [12,15]. This same trend was consistent with these populations. The percentage of the three predominant fatty acids (C16:0, C18:1, and C18:2) detected in the extracted oil in these populations ranged from 84 to 93.75%.

Tukey’s method was utilized to compare which *ahFAD2* genotypes had significantly different means for each fatty acid collected in the entire population (Table 4). In the whole population, the mean values of stearic and behenic acids were not significantly different by genotype; but, the means of the remaining six fatty acids did vary by genotype. Comparing each of the nine genotypes detected along with their respective oleic acid mean demonstrated that as the number of mutant alleles increased so did the total oleic acid content. However, the oleic acid content of *ahFAD2* genotypes with the same number of mutant alleles was not statistically different (Table 4). Therefore, the mean oleic values were equivalent for the following genotypes Ol_1_ol_1_Ol_2_ol_2_, ol_1_ol_1_Ol_2_Ol_2_, and Ol_1_Ol_1_ol_2_ol_2_. This same trend was observed for linoleic acid. The mean values of oleic and linoleic fatty acids sorted by the nine possible genotypes were only significantly different in the population when the number of mutant or wild type alleles increased or decreased (Table 4). This analysis revealed a total of five distinct phenotypes for the oleic and linoleic trait based on having 0, 1, 2, 3, or 4 mutant alleles in the *ahFAD2* genotype. This data suggests there is a dosage effect for the oleic and linoleic phenotypes in that as the number of mutant alleles increases the oleic acid content increases and the linoleic acid content decreases. Further, this data suggests that mutant alleles in the A genome do not have a bigger effect on oleic acid accumulation than the mutant alleles in the B genome or vice versa. The largest effect in boosting the oleic content was achieved by increasing the total number of mutant alleles in the progeny. A similar trend was also observed for palmitic acid except that only four distinct phenotypes were observed. The mean for palmitic acid produced from the wild type genotype (Ol_1_Ol_1_Ol_2_Ol_2_) was not significantly different from the means of genotypes containing one *ahFAD2* mutant allele (Ol_1_ol_1_Ol_2_Ol_2_ or Ol_1_Ol_1_Ol_2_ol_2_). Nevertheless, as the number of mutant alleles (2, 3, & 4) increased in the progeny, the means of palmitic acid grouped by genotype were significantly different.

**Table 4:** The fatty acid mean comparison split into each of the genotypes detected from the six segregating populations

**Fatty acid**	**F Ratio and P value**	**Genotype**	**Mean**	**Tukey**	**Fatty acid**	**F Ratio and P value**	**Genotype**	**Mean**	**Tukey**
**Palmitic C16:0**	F=150 ; P < 0.0001	Ol_1_Ol_1_Ol_2_Ol_2_	11.87	A	**Arachidic 20:0**	F=2.39; P = 0.0154	Ol_1_Ol_1_Ol_2_Ol_2_	1.42	AB
		Ol_1_ol_1_Ol_2_Ol_2_	11.28	A			Ol_1_ol_1_Ol_2_Ol_2_	1.38	AB
		Ol_1_Ol_1_Ol_2_ol_2_	11.23	A			Ol_1_Ol_1_Ol_2_ol_2_	1.44	A
		Ol_1_ol_1_Ol_2_ol_2_	10.34	B			Ol_1_ol_1_Ol_2_ol_2_	1.42	A
		Ol_1_Ol_1_ol_2_ol_2_	10.35	B			Ol_1_Ol_1_ol_2_ol_2_	1.32	AB
		ol_1_ol_1_Ol_2_Ol_2_	10.15	B			ol_1_ol_1_Ol_2_Ol_2_	1.38	AB
		Ol_1_ol_1_ol_2_ol_2_	8.84	C			Ol_1_ol_1_ol_2_ol_2_	1.24	B
		ol_1_ol_1_Ol_2_ol_2_	8.33	C			ol_1_ol_1_Ol_2_ol_2_	1.37	AB
		ol_1_ol_1_ol_2_ol_2_	6.15	D			ol_1_ol_1_ol_2_ol_2_	1.30	AB
**Stearic 18:0**	F=1.49; P = 0.1560	Ol_1_Ol_1_Ol_2_Ol_2_	2.95	A	**Gadoleic 20:1**	F=40.92; P < 0.0001	Ol_1_Ol_1_Ol_2_Ol_2_	1.06	C
		Ol_1_ol_1_Ol_2_Ol_2_	3.00	A			Ol_1_ol_1_Ol_2_Ol_2_	1.08	C
		Ol_1_Ol_1_Ol_2_ol_2_	2.97	A			Ol_1_Ol_1_Ol_2_ol_2_	1.14	C
		Ol_1_ol_1_Ol_2_ol_2_	2.96	A			Ol_1_ol_1_Ol_2_ol_2_	1.19	C
		Ol_1_Ol_1_ol_2_ol_2_	2.66	A			Ol_1_Ol_1_ol_2_ol_2_	1.30	BC
		ol_1_ol_1_Ol_2_Ol_2_	2.82	A			ol_1_ol_1_Ol_2_Ol_2_	1.24	C
		Ol_1_ol_1_ol_2_ol_2_	2.53	A			Ol_1_ol_1_ol_2_ol_2_	1.44	B
		ol_1_ol_1_Ol_2_ol_2_	2.76	A			ol_1_ol_1_Ol_2_ol_2_	1.47	B
		ol_1_ol_1_ol_2_ol_2_	2.69	A			ol_1_ol_1_ol_2_ol_2_	1.95	A
**Oleic 18:1**	F=351; P < 0.0001	Ol_1_Ol_1_Ol_2_Ol_2_	42.02	E	**Behenic 22:0**	F=0.83; P = 0.5701	Ol_1_Ol_1_Ol_2_Ol_2_	3.21	A
		Ol_1_ol_1_Ol_2_Ol_2_	45.83	D			Ol_1_ol_1_Ol_2_Ol_2_	3.07	A
		Ol_1_Ol_1_Ol_2_ol_2_	45.50	D			Ol_1_Ol_1_Ol_2_ol_2_	3.28	A
		Ol_1_ol_1_Ol_2_ol_2_	51.43	C			Ol_1_ol_1_Ol_2_ol_2_	3.25	A
		Ol_1_Ol_1_ol_2_ol_2_	53.28	C			Ol_1_Ol_1_ol_2_ol_2_	3.11	A
		ol_1_ol_1_Ol_2_Ol_2_	50.60	C			ol_1_ol_1_Ol_2_Ol_2_	3.18	A
		Ol_1_ol_1_ol_2_ol_2_	62.70	B			Ol_1_ol_1_ol_2_ol_2_	3.03	A
		ol_1_ol_1_Ol_2_ol_2_	63.14	B			ol_1_ol_1_Ol_2_ol_2_	3.18	A
		ol_1_ol_1_ol_2_ol_2_	79.44	A			ol_1_ol_1_ol_2_ol_2_	3.08	A
**Linoleic 18:2**	F=383; P < 0.0001	Ol_1_Ol_1_Ol_2_Ol_2_	36.05	A	**Lignoceric 24:0**	F=5.00; P < 0.0001	Ol_1_Ol_1_Ol_2_Ol_2_	1.41	BC
		Ol_1_ol_1_Ol_2_Ol_2_	32.96	B			Ol_1_ol_1_Ol_2_Ol_2_	1.40	C
		Ol_1_Ol_1_Ol_2_ol_2_	32.97	B			Ol_1_Ol_1_Ol_2_ol_2_	1.47	BC
		Ol_1_ol_1_Ol_2_ol_2_	27.98	C			Ol_1_ol_1_Ol_2_ol_2_	1.44	C
		Ol_1_Ol_1_ol_2_ol_2_	26.48	C			Ol_1_Ol_1_ol_2_ol_2_	1.50	ABC
		ol_1_ol_1_Ol_2_Ol_2_	29.16	C			ol_1_ol_1_Ol_2_Ol_2_	1.44	BC
		Ol_1_ol_1_ol_2_ol_2_	18.74	D			Ol_1_ol_1_ol_2_ol_2_	1.48	BC
		ol_1_ol_1_Ol_2_ol_2_	19.14	D			ol_1_ol_1_Ol_2_ol_2_	1.63	AB
		ol_1_ol_1_ol_2_ol_2_	3.73	E			ol_1_ol_1_ol_2_ol_2_	1.56	A

Means were compared by employing the Tukey Kramer test.

ANOVA was employed on the combined data set of Crosses 17, 19, and 28 (Table 5). These three crosses were chosen for the analysis due to their large population sizes and because all nine genotypes were detected by *ahFAD2* genotyping. This data set exhibited significant effects from the background genotypes of the parents on most of the fatty acids, suggesting that other loci besides *ahFAD2A* and *ahFAD2B* are involved in the production of these fatty acids (Table 5). The *ahFAD2* genotype also played a significant role in influencing levels of palmitic, oleic, linoleic, gadoleic acids, and the O/L ratio (P < 0.01) in these populations, suggesting a significant pleiotropic effect. The genotype influenced arachidic and lignoceric acid contents as well (P < 0.05). The *ahFAD2* genotype however, did not have any significant effect on stearic acid or behenic acid. These results were similar to a previously published report except that arachidic and lignoceric acids were previously not found to be affected by genotype [25]. Significant interactions between the *ahFAD2A* and *ahFAD2B* loci were detected for palmitic, oleic, linoleic acids, and the O/L ratio (P < 0.01), suggesting epistasis between these homoeologous genes. Further, significant additive and dominant affects were observed for both loci for all fatty acids except stearic, arachidic, behenic, and lignoceric acids. Due to the significant positive and negative additive and dominance estimates detected for most of the fatty acids collected, dominance appears to be incomplete for alleles of the *ahFAD2* genes. Further, incomplete dominance was also apparent from the intermediate mean values of palmitic, oleic, and linoleic acid phenotypes of the heterozygous *ahFAD2B* (ol_1_ol_1_Ol_2_ol_2_) progenies in Cross 25 compared to the homozygous recessive and dominant genotypes (Additional file 1: Table S1, Figure 3).

**Table 5:** **Mean squares analysis of variance across three 15**:**1 crosses 17**, **19**, &**28**

	**Palmitic**	**Stearic**	**Oleic**	**Linoleic**	**Arachidic**	**Gadoleic**	**Behenic**	**Lignoceric**	**O/L ratio**
Cross	19.79**	17.66**	155.17**	79.36**	2.05**	0.90**	5.70**	0.30†	35.45*
Genotypes	66.08**	1.20	3410.13**	2529.33**	0.15*	1.50**	0.60	0.22*	1191.92**
A genome locus	81.22**	0.10	3980.22**	2989.27**	0.07	1.52**	0.780	0.19	2250.85**
B genome locus	80.87**	0.51	4273.82**	3268.41**	0.04	1.88**	0.29	0.22	1887.65**
AB interaction	5.27**	0.89	485.01**	372.87**	0.12	0.25*	0.21	0.03	1137.08**
Cross x genotype	3.34**	0.66	99.13**	67.02**	0.05	0.12	0.32	0.10	12.44
Cross x A locus	0.59	1.15	23.25	19.47	0.03	0.27*	0.54	0.27*	19.29
Cross x a1	0.16	0.81	5.55	11.86	0.03	0.25†	0.65	0.30†	27.57†
Cross x d1	1.04	1.56	38.86	24.97	0.04	0.25†	0.52	0.25	8.51
Cross x B locus	4.44**	1.00	40.14	28.57†	0.09	0.03	0.22	0.02	21.36†
Cross x a2	6.04**	0.06	63.01†	36.20†	0.00	0.05	0.19	0.00	37.85*
Cross x d2	1.40	1.71	10.38	12.22	0.17†	0.03	0.26	0.04	11.97
C2	−0.39±0.16*	0.87±0.13**	−1.70±0.69*	0.68±0.56	0.30±0.04**	−0.13±0.05**	0.31±0.10**	0.05±0.05	−1.23±0.47**
a1	−1.27±0.11**	−0.04±0.09	8.91±0.47**	−7.68±0.39**	−0.03±0.03	0.15±0.03**	−0.06±0.07	0.01±0.03	5.78±0.32**
d1	0.29±0.17†	0.04±0.14	−1.91±0.70**	2.00±0.57**	−0.03±0.04	−0.12±0.05*	−0.17±0.10	−0.09±0.05†	−4.97±0.48**
a2	−1.28±0.11**	−0.07±0.09	9.38±0.47**	−8.21±0.39**	−0.03±0.03	0.19±0.03**	−0.04±0.07	0.04±0.03	5.85±0.32**
d2	0.04±0.17	−0.10±0.14	−1.51±0.71*	1.38±0.58*	−0.01±0.04	0.01±0.05	0.11±0.11	0.07±0.05	−4.75±0.48**
(aa)12	−0.39±0.11**	−0.06±0.09	3.87±0.47**	−3.43±0.39**	−0.03±0.03	0.09±0.03*	−0.05±0.07	0.00±0.03	5.40±0.32**
(ad)12	0.11±0.17	−0.14±0.14	−0.53±0.71	0.50±0.58	−0.03±0.04	0.02±0.05	0.01±0.11	0.05±0.05	−4.58±0.48**
(da)12	0.10±0.17	−0.17±0.14	−1.32±0.70†	1.43±0.57*	−0.04±0.04	−0.02±0.05	−0.01±0.10	0.02±0.05	−4.88±0.48**
(dd)12	0.48±0.25†	0.29±0.21	−3.61±1.06**	2.68±0.87**	0.13±0.06*	−0.09±0.07	0.16±0.16	−0.03±0.07	4.00±0.72**
Error	1.21	0.81	21.77	14.49	0.07	0.11	0.48	0.11	10.09

†, *, ** Denote significant effects at P<0.10, P<0.05, and P<0.01, respectively.

### Nematode resistance in cross 25

Root knot nematode [*Meloidogyne arenaria* (Neal) Chitwood race 1] causes significant economic loss to the southeastern USA peanut crop each year. Resistant peanut lines, such as ‘Tifguard’ [32], have been developed to resist infection and help prevent significant yield loss. Tifguard also has been demonstrated to carry field resistance to TSWV and was initially developed by crossing ‘C-99R’ [33] with ‘COAN’ [34]. The population was advanced via single seed descent then the resulting progenies were phenotyped [32]. Molecular markers were previously developed to discern the presence or absence of the gene *Rma* for resistance to *M*. *arenaria*[31,35]. Marker assisted selection (MAS) was employed to detect the progeny from Cross 25 (PI 651853 Tifguard/PI 653717 York) that were nematode resistant, as well as high oleic based on *Rma* and *ahFAD2* genotyping. Out of 125 F_2_ progeny, 28 (22.4%) were homozygous for the resistance allele, 46 (36.8%) were heterozygous, and 51 (40.8%) were homozygous for the susceptible allele. Of the 28 progeny carrying the nematode resistant allele, 11 (39%) were also determined to be high oleic, based on *ahFAD2* genotyping and gas chromatography. Chi square analysis demonstrated that this dominant trait was neither consistent with monogenic (3:1) segregation nor with digenic (15:1) segregation; however, these results were consistent with a 9:7 ratio. Segregation distortion was a factor, however, for this marker because the genotypic distribution did not fit a 1:2:1 ratio. There was an excess of susceptible alleles in this population. Significant segregation distortion of *Rma* alleles was also observed in a previous study evaluating F_4_ and F_5_ progeny [31]. This distortion could have been caused either by errors in genotyping, recombination between the SSR marker and *Rma* gene, distorted genotypes prior to or after fertilization, or complexity due to introgression of this trait from a diploid ancestor. Future work will include inoculating these susceptible and resistant lines to check for resistance to root galling and egg mass.

## Conclusion

Development of lines with enhanced traits is often a lengthy process of breeding, selection, and phenotypic evaluation through multiple generations to obtain the desired trait stacking. This is especially true when plants have long generation times and need to mature fully or be evaluated as densely planted late-generation populations across multiple environments prior to evaluating the presence or absence of multiple traits of interest. In theory, marker-assisted selection can be utilized as an aid in the breeding process to expedite the identification of a particular genotype linked to a desired phenotype in very early stages of a plant’s development. The markers utilized in this study allowed the selection of the high oleic trait, as well as, nematode resistant lines which can now be used to advance only the desirable progenies/selections in our breeding program. Analyzing the *ahFAD2* genotypes and fatty acid compositions of these segregating peanut populations clearly demonstrated that the fatty acid contents are quantitative in nature although much of the variability in the predominant fatty acids (oleic, linoleic, and palmitic) is controlled by only two loci. Oleic and linoleic acids displayed five unique phenotypes based on the total number of mutant or wild type alleles in the genotype. Dominance was incomplete for *ahFAD2* and both homoeologous loci displayed significant additive effects. Further, the *ahFAD2* loci do exhibit pleiotropic interactions for palmitic, oleic, linoleic acid contents, and the O/L ratio. Fatty acid levels in these progeny were affected by the parental genotype, suggesting that other genes influence fatty acid levels in peanut. These data demonstrate that the high oleic trait is not totally controlled by dominant gene action.

## Materials and methods

### Plant materials

Seeds were obtained from the USDA-ARS Plant Genetic Resources Conservation Unit in Griffin, GA. Two seeds per entry were germinated by planting them in a metal food serving tray containing a 1:1 mixture of Metro-Mix 300 (Griffin Greenhouse and Nursery Supplies, Ball Ground, GA) and perlite. Plants were watered daily using an automatic watering system and daylight was extended by turning the greenhouse lights on from 6 to 11 pm. Greenhouse conditions were set to maintain temperatures between 21°C and 29.5°C. Emasculation of anthers from flowers of the female parents started about six weeks after planting. Non-emasculated flowers were removed every morning. Flowers from the male parents were selected in the morning and placed in vials with water which were placed in a refrigerator until the evening. Pollinations were performed between 6:30 and 8:00 pm. Plastic wire ties were used to mark the emasculated flowers and aid in identification of the desired pegs. F_1_ seeds were harvested 90 d after the last pollination except for in crosses with PI 565455 Chico [36] which were harvested 80 d after the last pollination. All harvested F_1_ seeds were planted and grown in the greenhouse. Plants in the F_1_ generation determined to be a product of self pollination via *ahFAD2* genotyping were eliminated, while the hybrids were allowed to self and produce F_2_ seed.

One hundred twenty five F_2_ seed were randomly selected from each of the following four peanut populations: Cross 17 (PI 652938 Florida-07 *A*. *hypogaea* L. [37]/PI 280688 *A*. *hypogaea* subsp. *hypogaea* var. *hirsuta* Köhler), Cross 19 (PI 653717 York *A*. *hypogaea* L./PI 502096 *A*. *hypogaea* subsp. *fastigiata* Waldron var. *peruviana* Krapov. & W.C. Gregory), Cross 25 (PI 651853 Tifguard *A*. *hypogaea* L. [32]/PI 653717 York *A*. *hypogaea* L.), and Cross 28 (PI 565455 Chico *A*. *hypogaea* L. [36]/PI 653717 York *A*. *hypogae*a L.). Two smaller populations with 18 individuals from Cross 21 (PI 652938 Florida-07 *A*. *hypogaea* L. [37]/PI 502096 *A*. *hypogaea* subsp. *fastigiata* var. *peruviana*) and 21 individuals from Cross 27 (PI 565455 Chico *A*. *hypogaea* L. [36]/PI 652938 Florida-07 *A*. *hypogaea* L. [37]) were also included in the analysis. These crosses were selected because the parents were either high oleic (York and Florida-07) or represent diverse germplasm with traits such as early maturity (Chico) or pest/disease resistance to nematodes, leaf spots, or tomato spotted wilt virus (TSWV), all of which are significant problems for peanut production in the southeastern USA. All of the parents and 539 individual F_2_ seeds were evaluated by *ahFAD2* SNP genotyping [23,24] and total fatty acid composition (palmitic C16:0, stearic C18:0, oleic C18:1, linoleic C18:2, arachidic C20:0, gadoleic C20:1, behenic C22:0, and lignoceric acid C24:0) was collected to assess each genotype and phenotype (Additional file 1: Table S1). Method used to analyze total fatty acid composition is described below. Further, a total of 15% of the F_2_ progeny were randomly selected and the *ahFAD2* genotyping was replicated to ensure accuracy. No genotyping errors were revealed in this replication.

### DNA extraction and PCR

All DNA samples were extracted by following the directions from an Omega-BioTek E.Z.N.A Plant DNA kit (Norcross, GA.). Leaf tissue or slices from single seeds (75-150 mg) were used to extract DNA. Samples were placed in a 2 mL micro-centrifuge tube along with two 3 mm tungsten carbide beads (Qiagen Valencia, CA.) and 600 μl of P1 buffer from the Omega-BioTek kit. Tissue was pulverized by a Retsch Mixer Mill 301 (Leeds, UK) at 30 Hz for three minutes. Extracts were quantified on a DyNA Quant 200 fluorometer from Hoefer Pharmacia Biotech (San Francisco, CA). In addition, all samples were loaded on a 1% agarose gel (stained with ethidium bromide) along with a Low DNA Mass™ Ladder from Invitrogen (Carlsbad, CA) to evaluate quantity and quality of each extraction. All samples were subsequently diluted to 10 ng/μl for Real-Time PCR.

Genotyping assays were as described previously [23,24]. Briefly, genome specific SNPs identified from sequencing wild progenitors of cultivated peanut for *ahFAD2* were incorporated in the probe/primer design to preferentially select the A genome when genotyping *ahFAD2A* or the B genome when genotyping *ahFAD2B*. All PCR reactions were performed in an ABI StepOne™ Real-time PCR machine using MicroAmp® fast optical 48-well plate and adhesive film seals (Applied Biosystems, Foster City, CA.). Each PCR run included non-template controls to ensure that reagents were free of contaminants. In addition, several positive controls were included in each run, such as F435 to represent the homozygous recessive mutant alleles (ol_1_ol_2_), normal oleate lines to represent the homozygous wild type dominant alleles (Ol_1_Ol_2_), and heterozygous F_1_ progeny. StepOne version 2.0 (Applied Biosystems) was utilized to analyze and score genotypes among parents and progeny using the default parameters.

### Nematode resistance marker

The SSR marker GM565 [originally described as pPGSseq17E3, [38]] was employed to test F_2_ progeny for nematode resistance. This marker produces a 208 bp product in resistant lines and 195 bp product in susceptible lines [31,35]. The forward primer sequence was 5′ TTT CCT TTC AAC CCT TCG TG 3′ and the reverse sequence was 5′ AAT GAG ACC AGC CCA AAA TGC 3′. The primers were synthesized by MWG Operon (Huntsville, AL). The total PCR volume was 10 μl and consisted of 2.55 μl of H_2_0, 1× PCR Buffer, 2.5 mM MgCl_2_, 0.2 mM dNTPs, 0.15 μM forward primer, 0.15 μM reverse primer, 0.1 U/μl of Taq polymerase, and 0.75 ng/μl of diluted template DNA. The PCR buffer, polymerase, and dNTPs were all obtained from Promega (Madison, WI). Cycling conditions consisted of 1 cycle at 94°C for 5 min for the initial denaturing, 38 cycles of 94°C for 1 min, 56°C for 30 sec, and 72°C for 1 min, 1 cycle of 72°C for 10 min for final extension followed by a 4°C hold for temporal storage. All PCRs were performed in a GeneAmp 9700 thermocycler (Applied Biosystems Foster City, CA). The resulting products were separated on a 4% agarose gel mixed in a 1:1 ratio with a high resolving agarose (MetaPhor Cambrex Rockland, ME) to a standard molecular grade agarose and stained with ethidium bromide for visualization.

### Oil extraction and gas chromatography

Fatty acid composition was determined on an Agilent 7890A (Agilent Technologies, Santa Clara, CA) gas chromatograph with a flame ionization detector (FID). Oil from a small amount (~75 mg) of ground peanut seed was extracted in 5 mL of heptane and transesterified to fatty acid methyl esters (FAMEs) with 500 μl of 0.5 N sodium methoxide. Peak separation was performed on a DB-225 capillary column (15 m × 0.25 mm i.d. with a 0.25 μm film) from Agilent Technologies. One microliter of prepared sample was injected at a 60:1 split ratio into the column maintained isothermally at 280°C. The inlet and detector were set at 280°C and 300°C, respectively. The carrier gas was helium set at a flow rate of 1 mL/min (38 cm/sec). Peaks were identified by comparison to a FAME standard mix RM-3 (Sigma-Aldrich, St Louis, MO). A total of eight fatty acids (palmitic, stearic, oleic, linoleic, arachidic, gadoleic, behenic, and lignoceric acid) were identified in each peanut sample.

### Data analysis

GraphPad Prism version 3.0 and JMP version 9.0 were employed to statistically analyze the data and to construct graphs. Correlations were determined by employing the Pearson correlation and calculating a two-tailed P value with 95% confidence intervals. One way ANOVA was utilized to test for significant differences among the mean values of oleic acid for each genotypic class. Chi-square analysis was employed to test the segregation patterns for the oleic acid trait and to test for segregation distortion. Analysis of variance was employed to determine the significance of genotypic effects. Additive and dominance contrasts were estimated across all genotypes.

## Competing interests

The authors declare that they have no competing interests.

## Authors’ contributions

N.A.B. extracted DNA, performed *ahFAD2* genotyping, summarized genotyping data, analyzed data, initiated design and concept of study, and drafted the manuscript. R.N.P. made all the crosses to produce F_1_ and F_2_ seed, initiated design and concept of study, and revised the manuscript. T.G.I. carried out the mean squares analysis, statistical analysis, and revised the manuscript. M.L.W. helped edit the manuscript and made suggestions on study design. All authors read and approved the final manuscript.

## Supplementary Material

Additional file 1: Table S1 Summary of *ahFAD2* genotypes detected in each population (Crosses 17, 19, 21, 25, 27 & 28), as well as the number of individuals detected per genotype, and the mean percentage of each fatty acid detected per genotype are included. The following fatty acids were collected for each individual palmitic C16:0, stearic C18:0, oleic C18:1, linoleic C18:2, arachidic C20:0, gadoleic C20:1, behenic C22:0, and lignoceric acid C24:0. The standard deviations are also listed below. Click here for file

## References

[B1] Kris-Etherton PM Hu FB Ros E Sabate J The role of tree nuts and peanuts in the prevention of coronary heart disease: multiple potential mechanisms J Nutr 2008 138 9 1746S 1751S 18716180 10.1093/jn/138.9.1746S

[B2] Griel AE Eissenstat B Juturu V Hsieh G Kris-Etherton PM Improved diet quality with peanut consumption J Am Coll Nutr 2004 23 6 660 668 10.1080/07315724.2004.10719408 15637214

[B3] Sabate J Ang Y Nuts and health outcomes: new epidemiologic evidence Am J Clin Nutr 2009 89 5 1643S 1648S 10.3945/ajcn.2009.26736Q 19321572

[B4] Dean LL Henrix KW Holbrook CC Sanders TH Content of some nutrients in the core of the core of the peanut germplasm collection Peanut Sci 2009 36 104 120 10.3146/PS07-103.1

[B5] Teres S Barcelo-Coblijn G Benet M Alvarez R Bressani R Halver JE Escriba PV Oleic acid content is responsible for the reduction in blood pressure induced by olive oil Proc Natl Acad Sci USA 2008 105 37 13811 13816 10.1073/pnas.0807500105 18772370 PMC2544536

[B6] Pelkman CL Fishell VK Maddox DH Pearson TA Mauger DT Kris-Etherton PM Effects of moderate-fat (from monounsaturated fat) and low-fat weight-loss diets on the serum lipid profile in overweight and obese men and women Am J Clin Nutr 2004 79 2 204 212 14749224 10.1093/ajcn/79.2.204

[B7] Parthasarathy S Khoo JC Miller E Barnett J Witztum JL Steinberg D Low density lipoprotein rich in oleic acid is protected against oxidative modification: Implications for dietary prevention of atherosclerosis Proc Natl Acad Sci USA 1990 87 3894 3898 10.1073/pnas.87.10.3894 2339129 PMC54010

[B8] Nestel P Noakes M Belling B Mcarthur R Clifton P Janus E Abbey M Plasma-Lipoprotein Lipid and Lp[a] Changes with Substitution of Elaidic Acid for Oleic-Acid in the Diet J Lipid Res 1992 33 7 1029 1036 1431582

[B9] Vassiliou EK Gonzalez A Garcia C Tadros JH Chakraborty G Toney JH Oleic acid and peanut oil high in oleic acid reverse the inhibitory effect of insulin production of the inflammatory cytokine TNF-alpha both in vitro and in vivo systems Lipids Health Dis 2009 8 25 10.1186/1476-511X-8-25 19558671 PMC2706835

[B10] Yu S Pan L Yang Q Min P Ren Z Zhang H Comparison of the Delta(12) fatty acid desaturase gene between high-oleic and normal-oleic peanut genotypes J Genet Genomics 2008 35 11 679 685 10.1016/S1673-8527(08)60090-9 19022202

[B11] Andersen PC Gorbet DW Influence of year and planting date on fatty acid chemistry of high oleic acid and normal peanut genotypes J Agric Food Chem 2002 50 5 1298 1305 10.1021/jf0113171 11853521

[B12] Moore KM Knauft DA The inheritance of high oleic acid in peanut J Hered 1989 80 252 253

[B13] Jung S Swift D Sengoku E Patel M Teule F Powell G Moore K Abbott A The high oleate trait in the cultivated peanut [*Arachis hypogaea* L.]. I. Isolation and characterization of two genes encoding microsomal oleoyl-PC desaturases Mol Genet Genomics 2000 263 5 796 805 10.1007/s004380000244 10905347

[B14] Cohn JS Oxidized fat in the diet, postprandial lipaemia and cardiovascular disease Curr Opin Lipidol 2002 13 1 19 24 10.1097/00041433-200202000-00004 11790959

[B15] Norden AJ Gorbet DW Knauft DA Young CT Variability in oil quality among peanut genotypes in the Florida breeding program Peanut Sci 1987 14 7 11 10.3146/i0095-3679-14-1-3

[B16] Jung S Powell G Moore K Abbott A The high oleate trait in the cultivated peanut [*Arachis hypogaea* L]. II. Molecular basis and genetics of the trait Mol Genet Genomics 2000 263 5 806 811 10.1007/s004380000243 10905348

[B17] López Y Nadaf HL Smith OD Connell JP Reddy AS Fritz AK Isolation and characterization of the Delta(12)-fatty acid desaturase in peanut (*Arachis hypogaea* L.) and search for polymorphisms for the high oleate trait in Spanish market-type lines Theor Appl Genet 2000 101 7 1131 1138 10.1007/s001220051589

[B18] Ray TK Holly SP Knauft DA Abbott AG Powell GL The primary defect in developing seed from the high oleate variety of peanut (*Arachis hypogaea* L.) is the absence of delta 12-desaturase activity Plant Sci 1993 91 15 21 10.1016/0168-9452(93)90184-2

[B19] Schwartzbeck JL Jung S Abbott AG Mosley E Lewis S Pries GL Powell GL Endoplasmic oleoyl-PC desaturase references the second double bond Phytochemistry 2001 57 5 643 652 10.1016/S0031-9422(01)00081-4 11397429

[B20] Bruner AC Jung S Abbott AG Powell GL The naturally occurring high oleate oil character in some peanut varieties results from reduced oleoyl-pc desaturase activity from mutation of aspartate 150 to asparagine Crop Sci 2001 41 522 526 10.2135/cropsci2001.412522x

[B21] López Y Smith OD Senseman SA Rooney WL Genetic factors influencing high oleic acid content in Spanish market-type peanut cultivars Crop Sci 2001 41 1 51 56 10.2135/cropsci2001.41151x

[B22] Chu Y Ramos L Holbrook CC Ozias-Akins P Frequency of a loss-of-function mutation in oleoyl-PC desaturase (ahFAD2A) in the mini-core of the US peanut germplasm collection Crop Sci 2007 47 6 2372 2378 10.2135/cropsci2007.02.0117

[B23] Barkley NA Chamberlin Chenault KD Wang ML Pittman RN Development of a real-time PCR genotyping assay to identify high oleic acid peanuts (*Arachis hypogaea* L.) Mol Breed 2010 25 541 548 10.1007/s11032-009-9338-z

[B24] Barkley NA Wang ML Pittman RN A real-time PCR genotyping assay to detect FAD2A SNPs in peanuts (Arachis hypogaea L.) Electron J Biotechnol 2011 10.2225/vol14-issue1-fulltext-12

[B25] Isleib TG Wilson RF Novitzky WP Partial dominance, pleiotropism, and epistasis in the inheritance of the high oleate trait in peanut Crop Sci 2006 46 1331 1335 10.2135/cropsci2005.09-0313

[B26] Barkley NA Chamberlin Chenault KD Wang ML Pittman RN Genotyping and fatty acid composition analysis in segregating peanut (Arachis hypogaea L.) populations Peanut Sci 2011 38 1 11 19 10.3146/PS10-17.1

[B27] Isleib TG Young CT Knauft DA Fatty acid genotypes of five Virginia-type peanut cultivars Crop Sci 1996 36 556 558 10.2135/cropsci1996.0011183X003600030003x

[B28] Zhu C Zhang YM An EM algorithm for mapping segregation distortion loci BMC Genet 2007 8 82 18047652 10.1186/1471-2156-8-82 PMC2257974

[B29] Hong Y Chen X Liang X Liu H Zhou G Li S Wen S Holbrook CC Guo B A SSR-based composite genetic linkage map for the cultivated peanut (Arachis hypogaea L.) genome BMC Plant Biol 2010 10 17 10.1186/1471-2229-10-17 20105299 PMC2835713

[B30] Varshney RK Bertioli DJ Moretzsohn MC Vadez V Krishnamurthy L Aruna R Nigam SN Moss BJ Seetha K Ravi K The first SSR-based genetic linkage map for cultivated groundnut (Arachis hypogaea L.) Theor Appl Genet 2009 118 4 729 739 10.1007/s00122-008-0933-x 19048225

[B31] Nagy ED Chu Y Guo Y Khanal S Tang S Li Y Dong W Timper P Tayler C Ozias-Akins P Recombination is suppressed in an alien introgression on chromosome 5A of peanut harboring Rma, a dominant root knot nematode resistant gene Mol Breed 2010 26 357 370 10.1007/s11032-010-9430-4

[B32] Holbrook CC Timper P Culbreath AK Kvien CK Registration of ‘Tifguard’ peanut J Plant Reg 2008 2 2 92 94 10.3198/jpr2007.12.0662crc

[B33] Gorbet DW Shokes FM Registration of ‘C-99R’ peanut Crop Sci 2002 42 2207 10.2135/cropsci2002.2207

[B34] Simpson CE Starr JL Registration of ‘COAN’ peanut Crop Sci 2001 41 918 10.2135/cropsci2001.413918x

[B35] Chu Y Wu CL Holbrook CC Tillman BL Person G Ozias-Akins P Marker assisted selection to pyramid nematode resistance and the high oleic trait in peanut Plant Genome 2011 4 2 110 117 10.3835/plantgenome2011.01.0001

[B36] Bailey WK Hammons RO Registration of Chico peanut germplasm (Reg. No. GP 2) Crop Sci 1975 15 1 105

[B37] Gorbet DW Tillman BL Registration of ‘Florida-07’ peanut J Plant Reg 2009 3 1 14 18 10.3198/jpr2008.05.0276crc

[B38] Ferguson ME Burow MD Schulze SR Bramel PJ Paterson AH Kresovich S Mitchell S Microsatellite identification and characterization in peanut (A-hypogaea L.) Theor Appl Genet 2004 108 6 1064 1070 10.1007/s00122-003-1535-2 15067392

