# Supplementary material for: Genotypic effect of ahFAD2 on fatty acid profiles in six segregating peanut (Arachis hypogaea L) populations
Source: BMC Genet. 2013 Jul 17;14:62. doi: 10.1186/1471-2156-14-62 (PMC3722029; doi:10.1186/1471-2156-14-62)
Supplement: Additional file 1: Table S1 — Summary of ahFAD2 genotypes detected in each population (Crosses 17, 19, 21, 25, 27 & 28), as well as the number of individuals detected per genotype, and the mean percentage of each fatty acid detected per genotype are included. The following fatty acids were collected for each individual palmitic C16:0, stearic C18:0, oleic C18:1, linoleic C18:2, arachidic C20:0, gadoleic C20:1, behenic C22:0, and lignoceric acid C24:0. The standard deviations are also listed below. [file 1471-2156-14-62-S1.pdf]

Supplementary Table 1: Summary of *ahFAD2* genotypes detected in each population (Crosses 17, 19, 21, 25, 27 & 28), as well as the number of individuals detected per genotype, and the mean percentage of each fatty acid detected per genotype are included. The following fatty acids were collected for each individual palmitic C16:0, stearic C18:0, oleic C18:1, linoleic C18:2, arachidic C20:0, gadoleic C20:1, behenic C22:0, and lignoceric acid C24:0. The standard deviations are also listed below.

**F2 Population**	**Count**	***ahFAD2* Genotype**	**16:0**	**18:0**	**18:1**	**18:2**	**20:0**	**20:1**	**22:0**	**24:0**	**O/L**
17	09	$Ol_1Ol_1Ol_2Ol_2$	13.20 ±1.52	2.25 ±0.62	40.47 ±4.77	37.72 ±2.91	1.19 ±0.22	1.13 ±0.28	2.71 ±0.66	1.34 ±0.39	1.09 ±0.21
17	22	$Ol_1Ol_1Ol_2ol_2$	11.63 ±1.02	2.61 ±0.89	45.68 ±3.49	33.24 ±2.91	1.31 ±0.22	1.20 ±0.30	2.92 ±0.47	1.42 ±0.33	1.39 ±0.23
17	13	$Ol_1ol_1Ol_2Ol_2$	12.08 ±0.93	2.88 ±0.82	47.14 ±5.08	32.42 ±4.54	1.26 ±0.20	0.89 ±0.29	2.30 ±0.49	1.04 ±0.25	1.50 ±0.37
17	15	$ol_1ol_1Ol_2Ol_2$	11.83 ±1.38	2.55 ±0.61	49.26 ±6.01	29.97 ±4.64	1.26 ±0.21	1.12 ±0.37	2.74 ±0.89	1.27 ±0.62	1.72 ±0.55
17	26	$Ol_1ol_1Ol_2ol_2$	10.87 ±1.41	2.78 ±1.17	52.64 ±4.98	27.39 ±4.49	1.32 ±0.37	1.12 ±0.42	2.61 ±0.82	1.26 ±0.45	2.01 ±0.54
17	11	$Ol_1Ol_1ol_2ol_2$	10.84 ±1.31	2.56 ±0.90	53.74 ±4.18	25.99 ±3.45	1.27 ±0.22	1.33 ±0.39	2.79 ±0.37	1.47 ±0.33	2.11 ±0.40
17	12	$ol_1ol_1Ol_2ol_2$	9.35 ±1.52	2.45 ±0.74	62.59 ±5.43	18.41 ±4.38	1.28 ±0.29	1.46 ±0.48	2.91 ±0.96	1.54 ±0.46	3.65 ±1.22
17	09	$Ol_1ol_1ol_2ol_2$	9.34 ±0.60	2.74 ±0.75	64.28 ±2.63	17.83 ±1.90	1.22 ±0.20	1.13 ±0.30	2.29 ±0.62	1.17 ±0.44	3.66 ±0.54
17	04	$ol_1ol_1ol_2ol_2$	7.07 ±1.32	2.17 ±0.67	79.74 ±5.69	4.64 ±4.22	1.12 ±0.23	1.55 ±0.43	2.40 ±0.40	1.33 ±0.25	27.97 ±17.07
			11.08 ±1.76	2.61 ±0.86	52.13 ±9.49	27.72 ±8.00	1.27 ±0.26	1.18 ±0.39	2.69 ±0.70	1.33 ±0.43	2.85 ±5.39
19	09	$Ol_1Ol_1Ol_2Ol_2$	11.57 ±1.32	2.45 ±0.88	43.67 ±5.85	34.96 ±5.42	1.25 ±0.21	1.20 ±0.26	3.40 ±0.50	1.50 ±0.31	1.31 ±0.42
19	17	$Ol_1ol_1Ol_2Ol_2$	10.79 ±1.60	2.68 ±1.17	49.10 ±6.72	30.27 ±5.51	1.26 ±0.27	1.25 ±0.37	3.25 ±0.71	1.41 ±0.35	1.74 ±0.68
19	07	$Ol_1Ol_1Ol_2ol_2$	10.26 ±1.00	2.51 ±0.63	51.16 ±5.60	28.66 ±4.36	1.28 ±0.24	1.30 ±0.30	3.35 ±0.60	1.49 ±0.23	1.85 ±0.51
19	09	$Ol_1ol_1Ol_2ol_2$	9.85 ±1.22	2.63 ±0.87	54.69 ±5.12	25.38 ±4.33	1.30 ±0.29	1.31 ±0.26	3.37 ±0.45	1.47 ±0.18	2.25 ±0.60
19	27	$ol_1ol_1Ol_2Ol_2$	9.13 ±0.90	2.54 ±0.82	55.86 ±4.07	25.21 ±3.27	1.25 ±0.24	1.38 ±0.36	3.17 ±0.69	1.47 ±0.16	2.27 ±0.44
19	12	$Ol_1Ol_1ol_2ol_2$	9.73 ±1.86	2.16 ±0.57	56.82 ±6.24	23.34 ±4.72	1.18 ±0.26	1.51 ±0.23	3.64 ±0.44	1.62 ±0.18	2.56 ±0.71
19	13	$Ol_1ol_1ol_2ol_2$	8.63 ±1.06	1.95 ±0.50	64.32 ±3.88	17.68 ±2.68	1.06 ±0.18	1.74 ±0.33	3.09 ±0.52	1.55 ±0.24	3.74 ±0.75
19	19	$ol_1ol_1Ol_2ol_2$	8.36 ±0.94	2.43 ±1.13	65.46 ±5.58	15.97 ±4.29	1.20 ±0.29	1.67 ±0.48	3.30 ±0.92	1.62 ±0.35	4.69 ±2.68
19	11	$ol_1ol_1ol_2ol_2$	6.84 ±0.62	2.52 ±1.55	79.19 ±1.87	3.66 ±1.27	1.16 ±0.30	2.01 ±0.54	3.12 ±0.75	1.50 ±0.34	25.47 ±13.46
			9.46 ±1.70	2.43 ±0.95	57.80 ±10.44	22.82 ±8.98	1.22 ±0.26	1.48 ±0.43	3.28 ±0.62	1.51 ±0.27	4.62 ±7.67
21	1	$Ol_1Ol_1Ol_2Ol_2$	11.16 ±0.00	3.33 ±0.00	41.84 ±0.00	36.80 ±0.00	1.74 ±0.00	0.75 ±0.00	3.09 ±0.00	1.30 ±0.00	1.14 ±0.00
21	3	$Ol_1ol_1Ol_2Ol_2$	11.62 ±1.48	2.02 ±0.96	40.78 ±6.45	38.38 ±5.29	1.08 ±0.30	1.35 ±0.43	2.94 ±0.15	1.85 ±0.38	1.09 ±0.34
21	1	$Ol_1Ol_1Ol_2ol_2$	11.64 ±0.00	2.25 ±0.00	41.55 ±0.00	38.17 ±0.00	1.11 ±0.00	1.01 ±0.00	2.85 ±0.00	1.43 ±0.00	1.09 ±0.00
21	5	$Ol_1ol_1Ol_2ol_2$	10.98 ±0.58	2.01 ±0.22	50.83 ±4.64	29.96 ±4.45	1.06 ±0.09	1.17 ±0.09	2.58 ±0.23	1.42 ±0.13	1.74 ±0.41
21	2	$ol_1ol_1Ol_2Ol_2$	10.28 ±1.00	1.74 ±0.12	55.58 ±7.31	25.81 ±7.25	0.98 ±0.00	1.43 ±0.28	2.76 ±0.52	1.45 ±0.26	2.28 ±0.93
21	1	$Ol_1Ol_1ol_2ol_2$	10.96 0.00	2.36 0.00	49.46 0.00	30.29 0.00	1.23 0.00	1.21 0.00	3.01 0.00	1.48 0.00	1.63 0.00
21	5	$Ol_1ol_1ol_2ol_2$	8.61 ±0.68	2.31 ±0.78	64.15 ±3.04	18.19 ±2.62	1.19 ±0.22	1.31 ±0.16	2.82 ±0.26	1.41 ±0.15	3.61 ±0.72
			10.40 ±1.40	2.17 ±0.62	52.29 ±9.78	28.49 ±8.50	1.14 ±0.23	1.24 ±0.24	2.79 ±0.27	1.49 ±0.24	2.14 ±1.11

25	24	$ol_1ol_1Ol_2Ol_2$	9.91 ±0.61	2.76 ±0.61	48.22 ±2.78	31.66 ±2.61	1.40 ±0.22	1.27 ±0.26	3.23 ±0.32	1.55 ±0.24	1.54 ±0.22
25	60	$ol_1ol_1Ol_2ol_2$	8.06 ±0.74	2.86 ±0.84	61.51 ±3.96	20.00 ±3.82	1.39 ±0.27	1.46 ±0.24	3.13 ±0.41	1.59 ±0.20	3.23 ±0.83
25	41	$ol_1ol_1ol_2ol_2$	5.75 ±0.37	2.63 ±0.84	80.02 ±1.66	3.53 ±0.99	1.28 ±0.24	2.03 ±0.42	3.03 ±0.51	1.73 ±0.28	24.32 ±6.39
			7.66 ±1.62	2.76 ±0.80	65.03 ±12.03	16.84 ±10.70	1.36 ±0.25	1.61 ±0.43	3.12 ±0.43	1.63 ±0.24	9.82 ±10.83
27	1	$Ol_1Ol_1Ol_2Ol_2$	10.66 ±0.00	2.45 ±0.00	40.97 ±0.00	38.54 ±0.00	1.44 ±0.00	1.07 ±0.00	3.24 ±0.00	1.63 ±0.00	1.06 ±0.00
27	4	$Ol_1ol_1Ol_2Ol_2$	11.37 ±1.05	2.68 ±1.28	40.92 ±3.11	37.40 ±3.00	1.43 ±0.62	1.12 ±0.41	3.27 ±0.42	1.83 ±0.20	1.10 ±0.17
27	4	$Ol_1ol_1Ol_2ol_2$	10.54 ±1.35	3.04 ±1.57	44.81 ±1.99	33.49 ±2.90	1.59 ±0.63	1.15 ±0.45	3.64 ±0.53	1.76 ±0.46	1.35 ±0.17
27	2	$Ol_1Ol_1ol_2ol_2$	10.08 ±0.61	2.69 ±0.43	49.41 ±3.20	31.35 ±2.52	1.30 ±0.03	1.03 ±0.04	2.74 ±0.40	1.41 ±0.10	1.59 ±0.23
27	4	$ol_1ol_1Ol_2Ol_2$	9.47 ±0.82	3.27 ±1.14	49.58 ±2.47	29.87 ±2.26	1.52 ±0.40	1.17 ±0.38	3.49 ±0.57	1.64 ±0.33	1.67 ±0.21
27	2	$Ol_1ol_1ol_2ol_2$	8.93 ±0.30	3.13 ±0.74	58.60 ±4.90	21.26 ±2.69	1.60 ±0.28	1.23 ±0.08	3.64 ±0.98	1.63 ±0.01	2.79 ±0.58
27	3	$ol_1ol_1Ol_2ol_2$	9.04 ±1.36	3.25 ±0.88	57.87 ±4.81	21.06 ±2.63	1.68 ±0.43	1.19 ±0.19	3.90 ±0.62	2.01 ±0.32	2.80 ±0.58
27	1	$ol_1ol_1ol_2ol_2$	5.38 ±0.00	3.52 ±0.00	78.40 ±0.00	3.34 ±0.00	1.95 ±0.00	1.33 ±0.00	4.15 ±0.00	1.92 ±0.00	23.47 ±0.00
			9.84 ±1.60	3.02 ±1.01	50.01 ±9.49	29.20 ±8.74	1.54 ±0.43	1.15 ±0.29	3.50 ±0.58	1.74 ±0.31	2.77 ±4.79
28	11	$Ol_1Ol_1Ol_2Ol_2$	11.20 ±1.13	3.94 ±0.82	42.08 ±6.20	35.28 ±5.38	1.72 ±0.24	0.91 ±0.16	3.48 ±0.73	1.39 ±0.23	1.27 ±0.58
28	15	$Ol_1Ol_1Ol_2ol_2$	11.21 ±0.69	3.62 ±0.80	42.95 ±3.10	34.35 ±2.49	1.67 ±0.23	1.02 ±0.22	3.66 ±0.53	1.52 ±0.21	1.26 ±0.19
28	20	$Ol_1ol_1Ol_2Ol_2$	11.03 ±0.82	3.76 ±1.35	43.31 ±2.99	34.21 ±2.63	1.67 ±0.41	1.01 ±0.24	3.53 ±0.77	1.48 ±0.29	1.28 ±0.18
28	31	$Ol_1ol_1Ol_2ol_2$	10.18 ±0.71	3.52 ±0.78	48.52 ±4.21	29.70 ±3.20	1.65 ±0.23	1.14 ±0.20	3.75 ±0.88	1.54 ±0.29	1.67 ±0.33
28	04	$Ol_1Ol_1ol_2ol_2$	10.07 ±0.75	3.88 ±0.30	48.71 ±4.47	29.93 ±4.47	1.72 ±0.14	1.05 ±0.06	3.25 ±0.56	1.39 ±0.10	1.68 ±0.44
28	11	$ol_1ol_1Ol_2Ol_2$	9.72 ±0.53	3.69 ±0.72	51.32 ±3.22	27.29 ±2.53	1.64 ±0.21	1.14 ±0.26	3.62 ±0.82	1.57 ±0.34	1.90 ±0.29
28	13	$Ol_1ol_1ol_2ol_2$	8.87 ±0.82	3.24 ±0.92	59.32 ±3.82	20.74 ±2.63	1.50 ±0.18	1.31 ±0.24	3.44 ±0.82	1.58 ±0.37	2.93 ±0.59
28	11	$ol_1ol_1Ol_2ol_2$	8.39 ±0.81	2.85 ±0.65	62.33 ±2.75	18.51 ±2.30	1.41 ±0.18	1.42 ±0.21	3.45 ±0.56	1.64 ±0.20	3.43 ±0.53
28	09	$ol_1ol_1ol_2ol_2$	6.77 ±0.50	3.33 ±0.85	77.06 ±1.78	4.35 ±1.37	1.55 ±0.22	1.76 ±0.41	3.48 ±0.64	1.71 ±0.32	19.99 ±8.61
			9.95 ±1.47	3.53 ±0.89	51.08 ±10.38	27.53 ±8.97	1.62 ±0.26	1.17 ±0.31	3.58 ±0.73	1.54 ±0.28	3.15 ±5.25
Total	539	All genotypes	9.51 ±2.03	2.86 ±1.00	56.60 ±11.95	23.67 ±10.18	1.32 ±0.47	1.32 ±0.50	3.18 ±0.75	1.51 ±0.51	5.11 ±8.14
